# Point Mutations in the 14-α Sterol Demethylase Cyp51A or Cyp51C Could Contribute to Azole Resistance in *Aspergillus flavus*

**DOI:** 10.3390/genes11101217

**Published:** 2020-10-17

**Authors:** Jose Lucio, Irene Gonzalez-Jimenez, Olga Rivero-Menendez, Ana Alastruey-Izquierdo, Teresa Pelaez, Laura Alcazar-Fuoli, Emilia Mellado

**Affiliations:** 1 Mycology Reference Laboratory, National Centre for Microbiology, Instituto de Salud Carlos III (ISCIII), Majadahonda, 28220 Madrid, Spain; jose.lucio@isciii.es (J.L.); irene.gonzalez@isciii.es (I.G.-J.); olgariveromenendez@gmail.com (O.R.-M.); anaalastruey@isciii.es (A.A.-I.); lalcazar@isciii.es (L.A.-F.); 2 Spanish Network for Research in Infectious Diseases (REIPI RD16/CIII/0004/0003), ISCIII, Majadahonda, 28220 Madrid, Spain; 3 Hospital Universitario Central de Asturias, Fundación para la Investigación Biosanitaria del Principado de Asturias (FINBA), Oviedo, 33011 Asturias, Spain; mtpelaez@gmail.com

**Keywords:** *Aspergillus flavus*, Cyp51s, azole resistance mechanisms, azole drugs, DMIs fungicides

## Abstract

Infections caused by *Aspergillus* species are being increasingly reported. *Aspergillus flavus* is the second most common species within this genus causing invasive infections in humans, and isolates showing azole resistance have been recently described. *A. flavus* has three *cyp*51-related genes (*cyp*51A, *cyp*51B, and *cyp*51C) encoding 14-α sterol demethylase-like enzymes which are the target of azole drugs. In order to study triazole drug resistance in *A. flavus*, three strains showing reduced azole susceptibility and 17 azole susceptible isolates were compared. The three *cyp*51-related genes were amplified and sequenced. A comparison of the deduced Cyp51A, Cyp51B, and Cyp51C protein sequences with other protein sequences from orthologous genes in different filamentous fungi led to a protein identity that ranged from 50% to 80%. Cyp51A and Cyp51C presented several synonymous and non-synonymous point mutations among both susceptible and non-susceptible strains. However, two amino acid mutations were present only in two resistant isolates: one strain harbored a P214L substitution in Cyp51A, and another a H349R in Cyp51C that also showed an increase of *cyp*51A and *cyp*51C gene expression compared to the susceptible strain ATCC2004304. Isolates that showed reduced in vitro susceptibility to clinical azoles exhibited a different susceptibility profile to demethylation inhibitors (DMIs). Although P214L substitution might contribute to azole resistance, the role of H349R substitution together with changes in gene expression remains unclear.

## 1. Introduction

*Aspergillus* spp. are opportunistic fungi that cause both allergic and invasive syndromes. The genus *Aspergillus* contains approximately 175 species, but only a few of them have been associated with human disease [1]. The primary route of infection is characterized by the inhalation of conidia which, due to their small size, easily reach the pulmonary alveoli and cause local and invasive infections [2]. Among the wide range of aspergillosis manifestations, invasive aspergillosis (IA) is the most severe one and is linked to high morbidity and mortality rates in immunocompromised patients [3,4,5]. Besides the risk of immunosuppressed patients to *Aspergillus* infections, sporadic cases of invasive aspergillosis on immunocompetent hosts have also been reported [6,7].

In general, aspergillosis is mainly caused by *Aspergillus fumigatus*, followed by *A. flavus*, *Aspergillus terreus, Aspergillus niger* and *Aspergillus nidulans* [8]. *A. flavus* has a wide environmental distribution favored by the formation of conidia that are highly tolerant to adverse conditions and is considered an important pathogen that is responsible for several diseases in humans [9]. In Spain, the FILPOP study reported *A. fumigatus* as the most frequently isolated species (48.5%)*,* followed by *A. flavus* (8.4%), *A. terreus* (8.1%), *Aspergillus tubingensis* (6.8%), and *A. niger* (6.5%) [10]. An updated study in 2018 showed an increase in the frequency of isolation of *A. fumigatus* (52.74%), followed by *A. niger* (5.27%), *A. flavus* (5.07%), and *A. terreus* (4.67%) [11]. These species distribution can vary from country to country. For instance, *A. flavus* is more prevalent in Asia, Africa and the Middle East and it is considered the most frequent species causing aspergillosis in those areas [12]. Moreover, outbreaks associated with *A. flavus* appear to be produced by single or closely related strains, in contrast to those associated with *A. fumigatus* [9].

The first-line antifungal treatment against aspergillosis is broad-spectrum azole drugs, although polyenes and echinocandins are also included as alternative or salvage therapy, respectively [13]. Triazole drugs target the 14-α sterol demethylase (Cyp51), an enzyme that plays a key role in the ergosterol biosynthesis pathway [14]. However, the treatment of *Aspergillus* infections has recently become increasingly complicated because some species are developing resistance to antifungal drugs worldwide [15]. In *A. fumigatus*, the development of azole resistance has been mainly attributed to two different routes: (i) an in vivo or in-host acquisition that can happen after prolonged azole therapy and (ii) an acquisition in the environmental setting due to use of demethylation inhibitors (DMIs) fungicides which have a similar structure to clinical azoles implying cross-resistance between both of them [16,17]. Besides the clinical use of antifungals to treat *A. flavus* in human infections, the use of azoles in agriculture raises the concern about *A. flavus* azole resistance related to the use of this class of antifungals in the fields since it is also an important crop associated pathogen [18]. Fungicides are traditionally used to avoid food contaminations, such as *A. flavus* and other fungi that can be harmful for humans due to their ability to produce large amounts of mycotoxins [19].

In contrast to *A. fumigatus,* information regarding antifungal resistance mechanisms in *A. flavus* is relatively scarce. *A. fumigatus* has two Cyp51 isoenzymes, Cyp51A and Cyp51B [20]. Azole resistance in this fungus has been associated with the presence of several *cyp*51A point mutations and variations in the promoter [16]. An interesting difference between *A. flavus* and other *Aspergillus* species is the existence of a third Cyp51 protein, the Cyp51C [21]. Recently, *A. flavus* isolates with in vitro azole resistance have been described and azole resistance mechanisms in these strains are being analyzed. To date, different *A. flavus* Cyp51 point mutations have been described [22,23,24,25,26], however their role in azole resistance cannot be assumed since most of them were eventually described in azole susceptible strains. To date, only one point mutation (Y319H) in *A. flavus* Cyp51C seems to be related to azole resistance although it needs further confirmation [25].

The objective of this work was to sequence the three Cyp51 paralogues in several azole susceptible and resistant *A. flavus* clinical strains and to analyze the expression of the three genes during fungal growth. We also analyzed if the specific mutations found in each Cyp51 protein could be responsible for their resistance phenotype to azole drugs. The susceptibility of these *A. flavus* strains to different imidazoles and triazoles commonly used in crop protection was also tested and the hypothetical link between the development of resistance in agriculture and in the clinical setting for *A. flavus* isolates is discussed.

## 2. Materials and Methods

### 2.1. Strains and Molecular Identification

Twenty *A. flavus* isolates obtained from clinical samples were analyzed (Table 1). Fourteen strains belonged to the Mycology Reference Laboratory collection (CM) and six strains were obtained from the Hospital Universitario Central de Asturias (TP). Sample origin and year of isolation of all the *A. flavus* isolates are displayed in Supplementary Table S1. *A. flavus* ATCC2004304 was used as reference strain. Conidia from each strain were cultured in GYEP liquid medium (0.3% yeast extract, 1% peptone; Difco, Soria Melguizo, Madrid, Spain) with 2% glucose (Sigma-Aldrich Química, Madrid, Spain) for 24 h at 37 °C. After mechanical disruption of the mycelium by vortex-mixing with glass beads, genomic DNA of isolates was extracted using the phenol-chloroform method [27]. Molecular identification was performed by PCR amplifying and sequencing ITS1-5.8S-ITS2 regions and a portion of the β-tubulin gene [28].

### 2.2. Antifungal Susceptibility Testing (AFST)

Microdilution testing was performed according to the European Committee on Antimicrobial Susceptibility Testing (EUCAST) standard methodology [29,30]. *A. fumigatus* ATCC2004305 and *A. flavus* ATCC2004304 were used as quality control strains. Antifungal agents used in the study were amphotericin B (AmB) (Sigma-Aldrich Química, Madrid, Spain), itraconazole (ITC) (Janssen Pharmaceutica, Madrid, Spain), voriconazole (VRC) (Pfizer SA, Madrid, Spain), posaconazole (POS) (Schering-Plough Research Institute, Kenilworth, NJ, USA), isavuconazole (ISV) (Basilea Pharmaceutica, Basel, Switzerland), terbinafine (TBR) (Novartis, Basel, Switzerland), caspofungin (Merck & Co., Inc., Rahway, NJ, USA), micafungin (Astellas Pharma, Inc., Tokyo, Japan), and anidulafungin (Pfizer SA, Madrid, Spain). The final concentrations tested ranged from 0.03 to 16 mg/L for amphotericin B, terbinafine, and caspofungin; from 0.015 to 8 mg/L for itraconazole, voriconazole, and posaconazole; from 0.008 to 4 mg/L for anidulafungin; and from 0.004 to 2 mg/L for micafungin. Microdilution plates were incubated at 35 °C for 48 h in a humid atmosphere. Visual readings were performed at 24 and 48 h with the help of a mirror. The endpoint for amphotericin B, itraconazole, voriconazole, posaconazole, isavuconazole, and terbinafine was the antifungal concentration that produced a complete inhibition of visual growth at 24 and 48 h (MIC). For the echinocandins, the endpoint was the antifungal concentration that produced a visible change in the morphology of the hyphae compared with the growth control well (minimum effective concentration (MEC)). EUCAST has set breakpoints for the interpretation of antifungal susceptibility testing results, although they have only been set for itraconazole and isavuconazole for *A. flavus* strains (MICs of ≤1 μg/mL susceptible and >1–2 Resistant). EUCAST breakpoint values set for other antifungals for *A. fumigatus* strains were used as a reference but taking into account that the epidemiological cut-off values (ECOFFs) [31] established for *A. flavus* strains are one dilution higher than for *A. fumigatus*: 0.5 mg/L for posaconazole (POS), and 4 mg/L for voriconazole (VRC). Strains were selected for further study if they were ITC resistant (ITC MIC >1 mg/L).

### 2.3. Susceptibility Testing to 14-α Sterol Demethylase Inhibitors (DMIs)

Two imidazole drugs, prochloraz and imazalil, and five triazole drugs, metconazole, tebuconazole, epoxiconazole, difenoconazole, bromuconazole and myclobutanil (Sigma-Aldrich Quimica SA, Madrid, Spain), that belong to the 14-α sterol demethylation inhibitors (DMIs) and are frequently used to protect crops, were tested following the EUCAST methodology. The final concentrations of each azole drug tested ranged from 0.06 to 32 mg/L. The plates were incubated at 35 °C for 48 h in a humid atmosphere. Visual readings were performed at 24 and 48 h with the help of a mirror. The endpoint for all of them was the antifungal concentration that produced a complete inhibition of visual growth at 24 and 48 h (MIC).

### 2.4. PCR Amplification and Sequence Analysis of the cyp51A, cyp51B and cyp51C Genes

The *cyp*51A, *cyp*51B and *cyp*51C genes, including their promoter region, were amplified and sequenced using the primers described in Supplementary Table S2. PCR conditions for amplification of each *cyp*51 gene were described before [32] although the annealing temperature varied depending on the primer combination. Specific conditions for *cyp*51A amplification were 1 cycle of 5 min at 94 °C and then 35 cycles of 30 s at 94 °C, 45 s at 54 °C for F1 and R1, 55 °C for F2 and R2, F3 and R3, F4 and R4, 2 min at 72 °C and, finally, 5 min at 72 °C. Specific conditions for *cyp*51B amplification were 1 cycle of 5 min at 94 °C and then 35 cycles of 30 s at 94 °C, 45 s at 60 °C for F1, and R4, 2 min at 72 °C and, finally, 5 min at 72°C. The parameters used for *cyp*51C amplification were 1 cycle of 5 min at 94 °C and then 35 cycles of 30 s at 94 °C, 45 s at 58 °C for 1_F and 2_R, 54 °C for 3_F and 4_R, 52 °C for 5_F and 6_R, 54 °C for 7_F and 8_R, 2 min at 72 °C and, finally, 5 min at 72 °C. To exclude the possibility that any change identified in the sequences was due to PCR-induced errors, each isolate was independently analyzed twice.

### 2.5. RNA Isolation, Reverse Transcription and Quantitative PCR (RT-qPCR)

The *A. flavus* inocula were added to 100 mL of minimal medium broth [33] reaching a final concentration of 10^6^/mL total conidia and grown 18 h at 150 rpm at 37 °C. Then, mycelium was harvested using a funnel and miracloth paper (Calbiochem^R^, Merck Millipore, Spain). Mycelial samples were blot dried, frozen in liquid nitrogen, and ground to powder using a pestle and a mortar. RNA was isolated from mycelial powder by using an RNeasy plant minikit (Qiagen, Madrid, Spain) and following manufacturer’s instructions. cDNA was obtained through reverse transcription using the commercial ImProm-IITM Reverse Transcription System kit (Promega, Madison, WI, USA). The PCR reaction mixture was prepared following manufacturer’s instructions (1μg of cellular RNA, 1 µg/mL of the primers (dT), 4.5 µL of RNase-free water, 4 µL of reaction buffer ImProm-II (5×), 4 µL of MgCl_2_, 1 µL of dNTPs, 0.5 µL of the ribonucleases inhibitor, rRNasin^®^ (Promega, Madison, WI, USA), and 1 µL of reverse transcriptase, ImProm-IITM (Promega, Madison, WI, USA), in a total volume of 20 µL. The reverse transcription was performed in a GeneAmp PCR System 9700 (Applied Biosystems, Foster City, CA, USA) by using the following program parameters: 5 min at 25 °C, 60 min at 42 °C, and 15 min at 70 °C. cDNA was diluted in RNase-free water (1:5) for qPCR.

Quantitative PCR (qPCR) was performed to determine the differences in relative expression of *cyp*51 genes between azole resistant and azole susceptible *A. flavus* isolates. The *A. flavus* β-tubulin gene was used as housekeeping gene. Specific primers for the qPCR (Supplementary Table S2) were designed and PCR conditions were standardized and optimized for each primer pair. After cDNA synthesis, qPCR mixtures were carried out with SensiMix SYBR-Hi carboxy-X-rhodamine (Bioline, Barcelona, Spain). The PCR conditions were 10 min at 95 °C and 40 cycles of 10 s at 95 °C, 5 s at 58 °C, and 30 s at 72 °C. Each assay was conducted in triplicates with RNA isolated from two biological replicas. Fold changes of gene expression were calculated using the 2^(−ΔCt)^ method for individual time points and normalized to the β-tubulin gene [34]. Fold changes in gene expression were calculated relative to cyp51A of the ATCC20044304 strain.

### 2.6. Sequence Computer Analysis

All sequence analyses were carried out using DNAstar software package, DNASTAR™ Lasergene Genomics Suite Software, (DNASTAR, Inc., Madison, WI, USA). Nucleotide sequences were analyzed using EditSeq and SeqMan and the amino acid sequences of the putative 14-α demethylase Cyp51A, Cyp51B, and Cyp51C proteins were deduced from nucleotide sequences using SeqBuilder.

### 2.7. Phylogenetic Analysis

Phylogenetic trees were obtained by maximum likelihood analysis [35] using InfoQuest^TH^ FP software, version 4.5 (Bio-Rad laboratories Inc, Hercules, CA, USA). Deduced proteins sequence of Cyp51A *A. flavus* NRRL3357(NCBI accession number XP_002375123); *A. flavus* AF70=MYA-384/AF70, (NCBI accession number KOC13200); *Aspergillus oryzae* RIB40 (NCBI accession number XP_001819419), Cyp51B *(A. flavus* NRRL3357 (NCBI accession number XP_002379130)*; A. flavus* AF70 (NCBI accession number KOC13803) and *A. oryzae* RIB40 (NCBI accession number XP_001822241) and Cyp51C *A. flavus* NRRL3357(NCBI accession number XP_002383931)*; A. flavus* AF70, (NCBI accession number KOC15064) and *A. oryzae* RIB40 (NCBI accession number XP_001824687) were compared with the amino acid sequences derived from Cyp51 proteins from other filamentous fungi, including other *Aspergillus* species, *Penicillium* spp., *Fusarium* spp., and other plant pathogens: *A. fumigatus* AfCyp51A; (NCBI accession number AAK73659); *A. fumigatus* AfCyp51B (NCBI accession number AAK73660); *A. terreus* AtCyp51A (NCBI accession number EAU33678); *A. terreus* AtCyp51B (NCBI accession number EAU36124); *Fusarium graminearum* FgCyp51A (NCBI accession number ESU09049); FgCyp51B (NCBI accession number EWZ31250) and FgCyp51C NCBI accession number ESU17718)*; Fusarium oxysporum* FoCyp51A (NCBI accession number RKK94077), FoCyp51B (NCBI accession number EWY82136) and FoCyp51C (NCBI accession number EWY82136); *Penicillium italicum* PiCyp51 (NCBI accession number CAA89824); *Penicillium digitatum* PdCyp51 (NCBI accession number CAD277993); *Oculimacula yallundae* OyCyp51 (NCBI accession number AAG44831); *Oculimacula acuformis* OaCyp51 (NCBI accession number AAF18468); *Neurospora crassa* NcCyp51 (NCBI accession number EAA34813.); *Erysiphe necátor* EnCyp51 (NCBI accession number AAD55135).

## 3. Results

### 3.1. Antifungal Susceptibility Testing

Using the EUCAST methodology, all *A. flavus* strains but three were susceptible to azole drugs tested, showing some strain-dependent variations (Table 1). Three strains (CM7668, CM8087 and CM9326) were considered resistant to azole drugs with three different susceptibility profiles. There were no major differences in susceptibility to amphotericin B (AmB) except for one strain (CM8087) that could be considered AmB resistant. No differences were found in susceptibility to either echinocandins or terbinafine (results not shown).

**Table 1:** Antifungal susceptibility profile of *A. flavus* isolates for various antifungal drugs. AmB, amphotericin B; ITC, itraconazole; VRC, voriconazole; POS, posaconazole; ISV, isavuconazole. In bold the *A. flavus* azole resistant strains. * Marked strains were previously tested in a preceding work [36].

Strains	MICs Ranges (mg/L)				
	AmB	ITC	VRC	POS	ISV
ATCC2004304	1	0.5	2	0.125	1
CM7668 *	0.5	>8	0.25	1–2	0.5
**CM8087**	8–>16	4–8	8	0.5	8
CM9165	1	0.25	0.5	0.125	0.25
CM9174	2	0.5–1	1–4	0.125–0.25	2–4
CM9189	1	0.5	1	0.25	1
CM9195	1	0.25	1	0.125	1
CM9228	1	0.5	0.5	0.25	2
CM9267	2	0.5	1	0.25	1
CM9298	1	0.5	1	0.125	1
CM9326 *	1–2	4–8	8	1–2	>8
CM9329	2	0.5	1	0.25	1
CM9331	1	0.5	1	0.25	2
CM9684	0.5	1	>8	0.25	2
TP642	1	1	2	0.25	2
TP968	1	1–2	4	0.5–1	4
TP992	1	0.5	2	0.25	1
TP1004	1	1	4	0.5	4
TP1115	1	1	4	0.5	4
TP1179	1	1	4	0.5	4

Note: isolates with ITC MIC > 1 mg/L were considered resistant and selected for futher studies.

### 3.2. Susceptibility Testing to 14-α Sterol Demethylase Inhibitors (DMIs) Fungicides

*A. flavus* susceptibility to each DMI antifungal drug is presented in Table 2. All strains but three exhibited MIC values to DMI fungicides similar to those of the *A. flavus* wild type strain (ATCC2004304), which is considered as a reference strain for EUCAST antifungal susceptibility testing. Two strains (CM8087 and CM9326) had higher MIC values to all imidazole and triazole drugs and one strain (CM7668) could be considered as hyper-susceptible to all DMIs. 

### 3.3. Sequence Analysis of cyp51A, cyp51B and cyp51C Genes

The three Cyp51 paralogues possess a different number of introns with different sizes. The GeneBank accession Numbers of all *A. flavus cyp51A*, *cyp51B* and *cyp51C* sequences are displayed in Supplementary Table S3.

#### 3.3.1. *Aspergillus flavus* Cyp51A

Gene *cyp*51A is 1591 bp long, with one intron of 67 bp (from base 194 to base 260) and that translates into 507 aa. The *cyp*51A sequences from strain ATCC2004304, *A. flavus* NRRL3357 (GenBank: XM_002375082.1), *A. flavus* AF70, and *A. oryzae* RIB40, were used as reference for comparison. The first 18 bp (atgatcttctcacgcagc) encoding six amino acid (MIFSRS) were excluded from the *A. flavus* NRRL3357 sequence because they did not match the other sequences (AF70 or RIB40). Changes in the *cyp*51A full coding sequence and derived amino acid sequences are displayed in Table 3. Several synonymous and non-synonymous point mutations were found among both susceptible and non-susceptible strains. One azole resistant strain (CM7668) harbored a c641t base change, resulting in a P214L point mutation that was only present in this strain, previously described in a recent work [36]. 

#### 3.3.2. *Aspergillus flavus* Cyp51B

Gene *cyp*51B is 1740 bp long, with three introns: the first one of 54 bp (from base 247 to base 300), the second one of 58 bp (from base 499 to base 556) and the last one of 53 bp (from base 1616 to base 1668) that translates into 524 aa. Cyp51B sequences from strain ATCC2004304, *A. flavus* NRRL3357, *A. flavus* AF70 and *A. oryzae* RIB40 were used as reference sequences. Changes in the *cyp*51B full coding sequence and derived amino acid sequences are displayed in Table 4. This protein only harbored four synonymous mutations and showed a fully conserved amino acid sequence in all azole susceptible and resistant *A. flavus* strains.

#### 3.3.3. *Aspergillus flavus* Cyp51C

Gene *cyp*51C is 1612 bp long, with one 70 bp intron (from base 197 to base 266) and translates into 513 aa. Cyp51C sequences from strain ATCC2004304, *A. flavus* NRRL3357, *A. flavus* AF70 and *A. oryzae* RIB40 were used as reference. Changes in the *cyp*51C full coding sequence and derived amino acid sequences are displayed in Table 5. Several synonymous and non-synonymous point mutations were found among both susceptible and non-susceptible strains. One azole resistant strain (CM9326) showed an a1046g base change resulting in a H349R point mutation that was only present in this strain, previously described in a recent work [36]. 

### 3.4. Aspergillus flavus cyp51 Genes Expression

**Figure 1:**
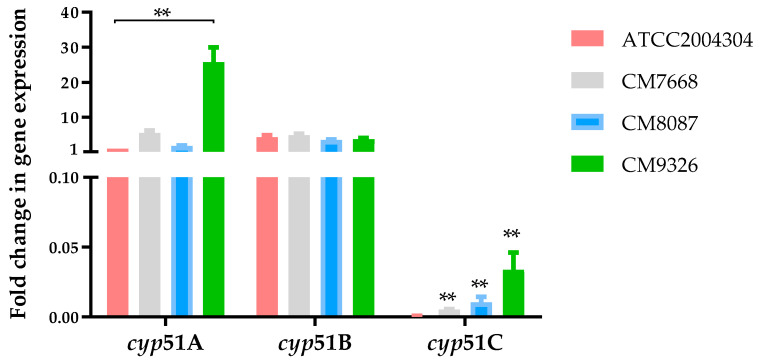
Fold change in gene expression of *cyp*51A, *cyp*51B and *cyp*51C in the ATCC2004304, CM7668, CM8087 and CM9326 *A. flavus* strains. Gene expression values are represented as bar plots with mean +SD. P-values were calculated using the Mann–Whitney U test, *p*-values of statistical tests are shown within the graphs. ** *p* < 0.001.

RT-PCR amplification was initially conducted to show that all *cyp*51 genes were expressed during hyphal growth (Supplementary Figure S1). Then, a quantitative PCR (qPCR) was performed to determine differences in relative expression of *cyp*51 genes between azole resistant (CM7668, CM8087 and CM9326) and the azole susceptible *A. flavus* ATCC2004304 strains (Figure 1). The expression of *cyp*51A was relatively similar between the strain ATCC2004304 and CM7668 and CM8087 strains. However, a significant increase of *cyp*51A expression was observed in the CM9326 isolate (*p* < 0.001). No differences of gene expression for the *cyp51B* were observed between all four isolates. Notably, the *cyp51C* gene was less expressed among all the strains (fold change of gene expression <500) compared to *cyp*51A or *cyp*51B. Although *cyp*51Cwas expressed in very low levels, we observed a significant increase of gene expression for all strains relative to the ATCC2004304 (Figure 1).

### 3.5. Sequence Analysis: Alignments and Similarity

The deduced 507 amino-acid protein encoded by the 1521-bp coding sequence of *A. flavus cyp*51A gene was compared to the known complete amino acid sequences of different fungal Cyp51 proteins including *Aspergillu*s species, *Penicillium* spp., *Fusarium* spp., and other plant pathogens. The strongest identities were shown with *A. terreus* Cyp51A (80.59%), *A. fumigatus* Cyp51A (77.60%), *A. flavus* Cyp51C (77%), *Penicillium* spp. Cyp51 (70%), *Fusarium* spp. Cyp51s (68 %), and *A. flavus* Cyp51B (58%). Homology with other plant pathogens, such as *N. crassa*, *O. yallundae, O. acuformis*, *E. necátor*, *Venturia inaequalis,* and *Venturia nashicola,* was around 58%.

The deduced 524 amino-acid protein encoded by the coding sequence of *A. flavus cyp*51B gene was compared to the known complete amino acid sequences of fungal Cyp51s. A strong identity was shown with *A. terreus* Cyp51B (84%), *A. fumigatus* Cyp51B (84%), *P. italicum* Cyp51 (78%), *Fusarium* spp. Cyp51s (63%), *A. flavus* Cyp51A (58%), and *A. flavus* Cyp51C (57%), while a range between 59% and 61% was shown with *N.crassa, O. yallundae, O. acuformis*, *E. necátor*, *V. inaequalis,* and *V. nashicola*.

The deduced 513-amino-acid protein encoded by the coding sequence *A. flavus cyp*51C gene was compared to the known complete amino acid sequences of fungal Cyp51s. A strong identity was shown with *A. fumigatus* Cyp51A (78%), *A. flavus* Cyp51A (77.56%), *A. terreus* Cyp51A (74.71%), *Penicillium* spp. Cyp51 (68%), *Fusarium* spp. Cyp51s (66%), *A. flavus* Cyp51B (60%), and *A. fumigatus* Cyp51B (60%). Identity to other plant pathogens ranged between 55% and 57%.

### 3.6. Phylogenetic Analysis

**Figure 2:**
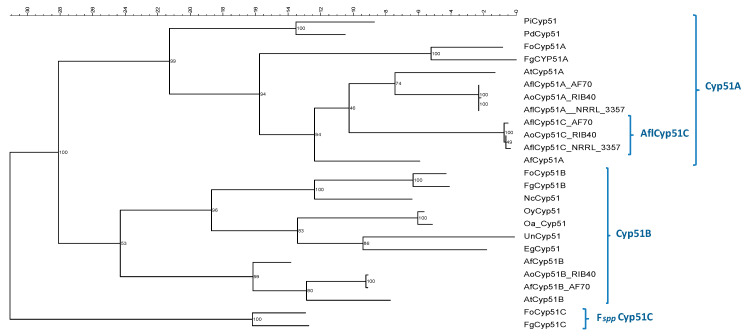
Phylogenetic tree obtained by maximum likelihood analysis. The bootstrap consensus tree was inferred from 2000 replicates to represent the phylogeny. The deduced amino acid sequence of the *A. flavus* Cyp51A, Cyp51B and Cyp51C; *A. oryzae* Cyp51A, Cyp51B and Cyp51C; *A. fumigatus* AfCyp51A and AfCyp51B; *A. terreus* AtCyp51A and AtCyp51B; *F. graminearum* FgCyp51A, FgCyp51B and FgCyp51C; *F. oxysporum* FoCyp51A, FoCyp51B and FoCyp51C; *P. italicum* PiCyp51 and *P. digitatum* PdCyp51; *O. yallundae* OyCyp51 and *O. acuformis* OaCyp51; *N. crassa* NcCyp51; *E. graminis* EgCyp51; and *U. necátor* UnCyp51.

The phylogenetic tree derived from the comparison of Cyp51′s protein sequences from *A. flavus* with those from other *Aspergillus* spp. and other fungal pathogens is shown in Figure 2. Well defined clusters could be detected for the Cyp51B-derived proteins and for the Cyp51A-derived proteins. However, the *A. flavus* Cyp51C sequences fall in a sub-cluster within the Cyp51A cluster. Interestingly, *Fusarium* spp. Cyp51C formed an independent cluster from Cyp51A or Cyp51B proteins.

## 4. Discussion

The emergence of *A. fumigatus* azole resistance is becoming an important issue worldwide [16]. Less frequently, cases of azole resistance in *A. flavus* have started to be described, originating a similar concern as *A. flavus* is the second most common *Aspergillus* spp., causing invasive infections in immunocompromised patients and the most frequent isolated species in some regions of the globe [12]. The extensive and prolonged use of azole drugs in immunocompromised patients has been reported as the main cause of the selection of azole resistant *A. fumigatus* strains [37]. Moreover, the agricultural industry uses pesticides, mainly azole fungicides, to optimize food production, and several studies have suggested an environmental route of *A. fumigatus* azole resistance through the exposure to azole fungicides in agriculture [38]. Although a similar phenomenon has never been described for *A. flavus,* this is an important issue because *A. flavus* produces aflatoxin which contaminates food and livestock feed resulting in significant annual crop losses internationally [39]. For this reason, fungicides, including azole fungicides (DMIs), are commonly used for crop protection [40,41]. Therefore, both the antifungal clinical use to treat *A. flavus* human infections and the use of azoles in agriculture raise the concern about *A. flavus* azole resistance emergence. Regardless of the route of development of azole resistance, the undesirable consequence of that event would be treatment failure [42].

Azole susceptibility in clinical and environmental *A. flavus* strains has been evaluated in several studies, although percentages of azole resistance are quite variable depending on the country and the study [12]. This variability could be due to the criteria used to establish susceptibility or resistance. According to EUCAST [31], *A. flavus* clinical breakpoints to azoles have only been established for itraconazole (ITC) (isolates are considered resistant with ITC MIC > 1 mg/L) and isavuconazole (ISV) (isolates are considered resistant with ISV MIC > 2 mg/L). ECOFFs are used for the rest of antifungals, and in general they are considered one step higher than for *A. fumigatus*: 0.5 mg/L for posaconazole (POS), and 4 mg/L for voriconazole (VRC). We considered azole resistance only if resistance to ITC was present. Considering these criteria in our study, only three of the *A. flavus* strains showed a reduced in vitro susceptibility to ITC. However, their susceptibility profile was quite different: the CM7668 strain was resistant to ITC and POS but not to VRC or ISV; strain CM9326 was resistant to all azole drugs and strain CM8087 was resistant to all azoles as well as Amphotericin B (AmB) (Table 1). Because azole resistance development has also been related to the use of DMIs in the field, we tested the most frequently used drugs to protect crops: imidazoles and triazoles (Table 2). Cross resistance between clinical azoles and DMIs was shown in the strains CM8087 and CM9326, which had elevated MICs to all the DMIs evaluated. However, the strain CM7668 showed lower MICs for all DMIs tested. This result indicates that different azole resistance mechanisms might be operating in these strains.

Following what has been established for *A. fumigatus*, one of the most studied species regarding amino acid substitutions in Cyp51 proteins, we studied the Cyp51 proteins in *A. flavus*. A key difference between *A. flavus* and other *Aspergillus* species is the existence of a third paralog in *A. flavus*, named *cyp*51C [24]. In contrast, other *Aspergillus* spp. and most filamentous fungi possess two Cyp51 proteins, Cyp51A and Cyp51B, although some species have only one [43]. Phylogenetic analysis shows that Cyp51B enzymes and all Cyp51s of those species with only one enzyme form a clear sub-group in the phylogenetic tree (Figure 2). Another subgroup contains all Cyp51As and includes *A. flavus* Cyp51C. This Cyp51C is relatively similar to *A. flavus* Cyp51A (77%) and it has been described in previous phylogenetic studies as a second Cyp51A [43]. The existence of this third paralog in *A. flavus* could explain why *A. flavu*s has higher MICs to all azole drugs, compared to *A. fumigatus*, as the inhibition of the three enzymes would be needed. Recent studies have shown that *Fusarium* spp. also have three Cyp51 paralogues (Cyp51A, -B, and -C), with Cyp51C being unique to the genus [44,45]. However, *Fusarium* spp. Cyp51C represents a complete different subgroup in the phylogenetic tree (Figure 2) [43]. In fact, it has recently been demonstrated that *Fusarium graminearum* Cyp51C does not encode a 14-α sterol demethylase [44].

In order to study azole drug resistance in *A. flavus*, the three genes encoding 14-α sterol demethylase enzymes (*cyp*51A, *cyp*51B, and *cyp*51C genes) were sequenced in all *A. flavus* strains included in this study and their deduced amino acid sequences were compared. We used the sequence of the *A. flavus* type strain (NRRL3357) as the reference sequence, because it was the first strain with the whole genome sequence [46]. Sequences from *A. flavus* (AF70) and *A. oryzae* (RIB40) were also included to see if the differences found between the sequences of the different Cyp51s in all the *A. flavus* clinical strains could correspond to very closely related species, such as *A. oryzae*, or different *A. flavus* morphotypes [47]. However, that was not the case. Several amino acid replacements were detected in Cyp51A and Cyp51C but none were found in Cyp51B. Previous studies have described different mutations in all three genes: *cyp*51A, *cyp*51B, and *cyp*51C. The initial studies [23] focused on mutations in Cyp51A and Cyp51B in spontaneous mutant strains obtained after voriconazole exposure. Although different mutations in both proteins were found (Y132N, K197N, D282E, M288L, and T469S in Cyp51A, and two combined mutations H399P/D411N and T454P/T486P in Cyp51B), these substitutions have been recently found in different *A. flavus* isolates regardless of their susceptibility to azoles, which makes their implication in drug resistance highly unlikely. Similarly, our Cyp51B results showed no amino acid changes in any of the *A. flavus* isolates, except for a few polymorphisms responsible for synonymous mutations (Table 4).

Cyp51A turns out to be highly polymorphic with several synonymous and non-synonymous point mutations (A199T, K332N, and T329A) that were found among azole susceptible and non-susceptible strains, therefore excluding their possible implication in azole resistance. However, one strain (CM7668) resistant only to ITC and POS harbored a c641t base change resulting in a P214L point mutation that was only present in this strain. This mutation is located at the equivalent position of P216L in *A. fumigatus* Cyp51A, a mutation that has been confirmed to be linked to azole resistance by gene replacement [48]. The amino acid P216 is a conserved amino acid within the fungal kingdom Cyp51s. Its role in azole resistance has been explored in Cyp51 homology models and results suggest that the alteration of this residue could be affecting the antifungal-enzyme interaction [49]. A previous homology model proved the interaction of P216L with POS but not with VRC, indicating that mutations in this region might play a role in POS resistance [49]. *A. fumigatus* strains with P216L mutation are resistant to itraconazole and posaconazole but not to voriconazole [50,51]. This peculiar azole susceptibility pattern is similar to the azole susceptibility phenotype exhibited by our *A. flavus* isolate which carries a P214L alteration. The location of the P214L substitution in Cyp51A of *A. flavus* strain CM7668, together with the similarity to the *A. fumigatus* azole susceptibility profile of strains with the P216L Cyp51A mutation, as well as the protein homology studies, highly suggest that this mutation is responsible for the azole resistance shown by this strain. Also, the hypersusceptibility to DMIs shown by this strain (Table 2) seems to indicate that the P214L mutation was not selected in the environment. A limitation of the present study was that clinical data were not collected and we cannot know if the patient was previously treated with ITC or POS, information which would be interesting in the study of the resistance mechanisms. Due to this lack of data, it is impossible to know if the development of azole resistance is related to an in-host acquisition or if this occurred before. However, previous reports *A. fumigatus* Cyp51A P214L mutated in patients treated with ITC will support this in vivo selection after ITC or POS treatment [48].

The other very polymorphic gene was *A. flavus cyp*51C with several synonymous and non-synonymous point mutations that were found among both susceptible and non-susceptible strains, and therefore excluding their possible implication in azole resistance. Several mutations found in this study were previously described in other studies, such as M54T, S240A, D254N, I285V, and N423D [24,25,26], while other substitutions are novel, including D254G, P276T, S399I, and a mutation responsible for a stop codon (R250_ST_). However, one strain (CM9326) resistant to all azole drugs harbored an a1064g base change resulting in a H349R point mutation that was only present in this strain (Table 5). How this mutation could be connected with the interaction of the azole drugs and the enzyme Cyp51C remains unclear. To date, only one point mutation (Y319H) in *A. flavus* Cyp51C suggests a relation to azole resistance although its role still needs further confirmation [25,26]. In fact, studies of homology modeling and molecular dynamic simulations showed its distant location from the iron porphyrin complex and its lack of effect on the docking of the azoles at the binding site and therefore its doubtful relevance in azole resistance [52].

Gene expression analysis (Figure 1) showed that *cyp*51A and *cyp*51B are predominantly expressed among all the strains compared to *cyp*51C, suggesting that 14-α sterol demethylase activity can be mainly attributed to Cyp51A and Cyp51B. These findings are supported by recent studies of the basal expressions of *A. flavus cyp*51s showed that *cyp*51A and *cyp*51B expression levels were greater than that of *cyp*51C, with an extremely low gene expression that can be strain-dependent [52]. Interestingly, the CM9326 strain showed higher gene expression levels of *cyp*51A and *cyp*51C than the reference strain ATCC2004304. Since the expression of *cyp*51C is at minimal detectable levels for all the strains, these results suggest an association between the *cyp*51A gene expression and the CM9326 azole resistance phenotype. Further experiments would be necessary to explore the role of the Cyp51C Y319H mutation in azole resistance alone or in combination with the *cyp*51A gene expression. Currently, one limitation of this study is that genetic manipulation of *A. flavus* is not fully developed yet, hence we cannot prove the implication of the Cyp51C Y319H mutation or increased Cyp51A expression in azole resistance.

One *A. flavus* strain (CM8087) with a susceptibility profile of resistance to all azoles did not show any amino acid change in any of the three enzymes, or their expression, that could explain or suggest an azole resistant mechanism. Other mechanisms, such as the upregulation of azole target (Cyp51s) or multidrug efflux transporters (ABC (ATP-binding cassette)/MFS (major facilitator superfamily)) that could lead to decreased drug concentrations within the fungal cell are starting to be explored [53]. The *cyp*51A, *cyp*51B, or *cyp*51C expression levels in *A. flavus* do not seem to be related to VRC resistance. In fact, the expression profiles of these genes barely vary among WT and non-WT strains, even after exposure to azoles [54]. Moreover, some studies on basal and voriconazole-induced expression of various efflux pump genes showed low basal expression irrespective of the azole susceptibility of the isolate [54]. Only a possible role of the multidrug efflux pump Cdr1B overexpression in *A. flavus* azole has been recently proposed [54]. Additionally, this strain (CM8087) was also resistant to AmB and therefore a different resistant mechanism would be expected. Further experiments will be needed to explore other resistance mechanisms that could explain this particular susceptibility profile. Cross resistance between azoles and AmB has been better analyzed in yeast. For instance, in *Candida tropicalis*, a bypass in the ergosterol biosynthesis pathway may develop as a result of mutations in sterol Δ5,6-desaturase (ERG3) together with Erg11/Cyp51 mutations, resulting in cross resistance to azoles and AmB [55]. In general, the ergosterol biosynthesis pathway is more complex in filamentous fungi and in particular, it has not been studied in *A. flavus* yet. Although, to date, it would be difficult to search for this kind of resistance mechanism in *A. flavus,* this possibility deserves consideration in further research.

## 5. Conclusions

In conclusion, antifungal resistance mechanisms in *A. flavus* isolates can be relevant in the clinical and in the environmental setting. The results of this study suggest that these mechanisms can be associated to *A. flavus* Cyp51A or Cyp51C mutations or are related to an increase of *cyp*51A gene expression. However, the role of these genes variations and their contribution to antifungal resistance needs to be further investigated.

## Figures and Tables

**Table 2:** Antifungal susceptibility profile of *A. flavus* isolates to imidazole and triazole demethylase inhibitors (DMIs). In bold the *A. flavus* azole resistant strains.

*A. flavus* strains	MICs Ranges (mg/L)							
	Imidazole		Triazole					
	Imazalil	Prochloraz	Metconazole	Tebuconazole	Epoxiconazole	Bromuconazole	Difenoconazole	Myclobutanil
ATCC2004304	1–2	2–4	4–8	8	8–16	4–16	8–>32	>32
**CM7668**	**0.25**	**1**	**1**	**1**	**0.5–1**	**0.25–1**	**0.5–1**	**4**
**CM8087**	**16**	**>32**	**>32**	**>32**	**>32**	**>32**	**>32**	**>32**
CM9165	2	1	8	8	8	8	16	>32
CM9174	4	2	16	16	32	8	16	>32
CM9189	2	1	8	8	8	8	16	>32
CM9195	2	1	8	4	8	8	16	>32
CM9228	2	1	8	4	8	8	16	32
CM9267	2	2	8	8	8	16	16	>32
CM9298	2	2	8	4	8	8	16	>32
**CM9326**	**32**	**>32**	**>32**	**>32**	**>32**	**>32**	**>32**	**>32**
CM9329	1	1	4	4	8	8	16	32
CM9331	2	1	8	8	16	8	32	>32
CM9684	8	2–4	16	16	16–>32	32–>32	16–>16	>32
TP642	8	8	16	16	>32	32	>32	>32
TP968	8	32	16	32	>32	16	32	>32
TP992	2	2	8	4	16	8	>32	>32
TP1004	8	8	16	16	>32	>32	>32	>32
TP1115	8	8	16	16	>32	>32	>32	>32
TP1179	8	8	16	32	>32	>32	>32	>32

**Table 3:** *Aspergillus flavus* Cyp51A analysis. The sequence of *A. flavus* NRRL3357 type strain was used as Reference. In bold the *A. flavus* azole resistant strains.

Strains	A *	*Aspergillus flavus* Cyp51A																
		c132t	c165t	c342t	g390a	t546c	g595a	c641t	t723c	c738t	t907c	t927c	g966t	a985g	c1065t	t1164c	c1368t	g1371a
		F44	P55	G114	K130	F182	A199T	P214L ^1^	Y241	N246	L303	P309	K322N	T329A	H355	P388	N456	L456
**CM7668**	**R**		c165t		g390a	t546c		**c641t**								t1164c		
**CM8087**	**R**		c165t		g390a	t546c								a985g		t1164c		
CM8098	S		c165t		g390a	t546c								a985g		t1164c		
CM9165	S		c165t			t546c			t723c							t1164c		
CM9174	S		c165t		g390a	t546c								a985g		t1164c		
CM9189	S		c165t		g390a	t546c										t1164c		
CM9195	S		c165t		g390a	t546c										t1164c		
CM9228	S					t546c					t907c		g966t			t1164c		
CM9267	S		c165t		g390a	t546c										t1164c		
CM9298	S	c132t	c165t	c342t	g390a	t546c						t927c				t1164c	c1368t	
**CM9326**	**R**		c165t		g390a	t546c										t1164c		
CM9329	S		c165t		g390a	t546c										t1164c		
CM9331	S		c165t		g390a	t546c										t1164c		
CM9684	S		c165t		g390a	t546c								a985g		t1164c		
TPH642	S		c165t		g390a	t546c										t1164c		
TPH968	S		c165t		g390a	t546c										t1164c		
TPH992	S		c165t		g390a	t546c										t1164c		
TPH1004	S		c165t		g390a	t546c										t1164c		
TPH1115	S		c165t		g390a	t546c										t1164c		
TPH1179	S		c165t		g390a	t546c										t1164c		
ATCC2004304	S		c165t		g390a	t546c	g595a								c1065t	t1164c	c1368t	g1371a
*A. oryzae* RIB40	-		c165t		g390a	t546c	g595a								c1065t	t1164c	c1368t	g1371a
*A. flavus* AF70	-		c165t		g390a	t546c				c738t						t1164c	c1368t	
**Protein**		**F44**	**P55**	**G114**	**K130**	**F182**	**A199T**	**P214L ^1^**	**Y241**	**N246**	**L303**	**P309**	**K322N**	**T329A**	**H355**	**P388**	**N456**	**L456**

* A, azole drugs profile, S susceptible, R Resistant. ^1^ This mutation was previously described in a recent work [36].

**Table 4:** *Aspergillus flavus* Cyp51B analysis. The sequence of *A. flavus* NRRL3357 was used as reference. In bold the *A. flavus* azole resistant strains.

Strains	A *	*Aspergillus flavus* Cyp51B			
		c237t	c498t	t699c	a799g
		C79	I166	N233	K267
**CM7668**	**R**				
**CM8087**	**R**				
CM9165	S	c237t	c498t		a799g
CM9174	S				
CM9189	S		c498t		
CM9195	S		c498t		
CM9228	S		c498t		
CM9267	S				
CM9298	S		c498t		
**CM9326**	**R**		c498t		
CM9329	S				
CM9331	S		c498t		
CM9684	S			t699c	
TPH642	S				
TPH968	S				
TPH992	S				
TPH1004	S				
TPH1115	S				
TPH1179	S				
ATCC2004304	S				
*A. oryzae* RIB40	-				
*A. flavus* AF70	-		c498t		a799g
Protein		C79	I166	N233	K267

* A, azole drugs profile, S susceptible, R, Resistant.

**Table 5:** *Aspergillus flavus* Cyp51C analysis. The sequence of A. flavus NRRL3357 (Type strain) was used as Reference. In bold the *A. flavus* azole resistant strains.

Strains	A *	*Aspergillus flavus* Cyp51C																		
		t161c	c174g	t718g	c748t	g760a	a761g	c826a	a853g	t876g	t894a	g915a	c978t	a1044g	a1046g	c1158t	g1196t	a1263g	a1267g	a1455g
		M54T	G58G	S240A	R250ST	D254N	D254G	P276T	I285V	A292A	S298S	M305I	L326	L348	H349R ^1^	S386	S399I	E421	N423D	V485V
**CM7668**	**R**	t161c	c174g	t718g			a761g						c978t	a1044g		c1158t		a1263g	a1267g	
**CM8087**	**R**	t161c	c174g	t718g																
CM9165	S	t161c	c174g	t718g																
CM9174	S	t161c	c174g	t718g																
CM9189	S	t161c	c174g	t718g			a761g	c826a	a853g	t876g	t894a					c1158t		a1263g	a1267g	
CM9195	S	t161c	c174g	t718g			a761g						c978t	a1044g		c1158t		a1263g	a1267g	
CM9228	S	t161c	c174g	t718g	c748t												g1196t			
CM9267	S	t161c	c174g	t718g			a761g						c978t	a1044g		c1158t		a1263g	a1267g	
CM9298	S	t161c	c174g	t718g			a761g						c978t	a1044g		c1158t		a1263g	a1267g	
**CM9326**	**R**	t161c	c174g	t718g											a1046g					
CM9329	S	t161c	c174g	t718g			a761g						c978t	a1044g		c1158t		a1263g	a1267g	
CM9331	S	t161c	c174g	t718g		g760a		c826a	a853g	t876g	t894a					c1158t		a1263g	a1267g	
CM9684	S	t161c	c174g	t718g																
TPH642	S	t161c	c174g	t718g			a761g						c978t	a1044g		c1158t		a1263g	a1267g	
TPH968	S	t161c	c174g	t718g			a761g						c978t	a1044g		c1158t		a1263g	a1267g	
TPH992	S	t161c	c174g	t718g			a761g						c978t	a1044g		c1158t		a1263g	a1267g	
TPH1004	S	t161c	c174g	t718g			a761g						c978t	a1044g		c1158t		a1263g	a1267g	
TPH1115	S	t161c	c174g	t718g			a761g						c978t	a1044g		c1158t		a1263g	a1267g	
TPH1179	S	t161c	c174g	t718g			a761g						c978t	a1044g		c1158t		a1263g	a1267g	
ATCC **	S	t161c	c174g	t718g		g760a			a853g	t876g	t894a					c1158t				a1455g
RIB40 ***	-	t161c	c174g	t718g																
AF70	-	t161c	c174g	t718g		g760a						g915a				c1158t		a1263g	a1267g	
**Protein**		**M54T**	**G58G**	**S240A**	**R250ST**	**D254N**	**D254G**	**P276T**	**I285V**	**A292A**	**S298S**	**M305I**	**L326**	**L348**	**H349R ^1^**	**S386**	**S399I**	**E421**	**N423D**	**V485V**

* A, azole drugs profile, S susceptible, R, Resistant. ** Reference strain *A. flavus* ATCC2004304. *** *A. oryzae.* ^1^ This mutation was previously described in a recent work [36].

## Supplementary Materials

The following are available online at https://www.mdpi.com/2073-4425/11/10/1217/s1, Table S1: Clinical *Aspergillus flavus* isolates: sample origin and year of isolation. Table S2: Primers used for *Aspergillus flavus cyp*51A, *cyp*51B and *cyp*51C DNA amplification and sequencing and RT-PCR from RNA. Table S3: GenBank Accession Numbers of all *A. flavus cyp*51A, *cyp*51B and *cyp*51C. Figure S1: *Aspergillus flavus cyp51* genes expression.
