# Supplementary material for: Point Mutations in the 14-α Sterol Demethylase Cyp51A or Cyp51C Could Contribute to Azole Resistance in Aspergillus flavus
Source: Genes (Basel). 2020 Oct 17;11(10):1217. doi: 10.3390/genes11101217 (PMC7602989; doi:10.3390/genes11101217)
Supplement: Supplementary file 1 [file genes-11-01217-s001.zip › genes-878464(1).pdf]

Supplementary Figure 1

Aspergillus flavus cyp51 genes expression

RT-PCR amplification was used to show that all three genes are expressed during hyphal growth in submerged culture. RNA isolation and the cDNA used in this amplification were obtained as indicated in section 2.5. RT-dependent products of the expected sizes were amplified for each of the *cyp*51 genes using a specific set of primers for each gene and that are displayed in Supplementary Table 2. The sequencing of the amplified bands from DNA and cDNA confirmed the presence of introns in each of the genes analyzed.

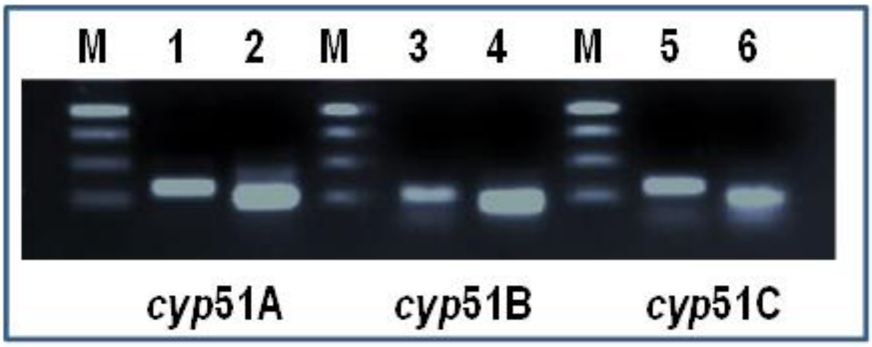

Figure 1. PCR and RT-PCR amplification of the three *A. flavus cyp*51 genes. *cyp*51A fragment from DNA (1) and cDNA (2); *cyp*51B fragment from DNA (3) and cDNA (4); *cyp*51C fragment from DNA (5) and cDNA (6). M; 1kb Ladder.
