# Supplementary material for: Point Mutations in the 14-α Sterol Demethylase Cyp51A or Cyp51C Could Contribute to Azole Resistance in Aspergillus flavus
Source: Genes (Basel). 2020 Oct 17;11(10):1217. doi: 10.3390/genes11101217 (PMC7602989; doi:10.3390/genes11101217)
Supplement: Supplementary file 1 [file genes-11-01217-s001.zip › genes-878464(2).pdf]

Supplementary Table 1. Clinical *Aspergillus flavus* isolates: sample origin and year of isolation. In red the *A. flavus* azole resistant strains.

Strains	Sample origin	Year Isolation
**CM7668**	Sputum	2015
**CM8087**	Cutaneous Biopsy	2016
**CM9165**	Bronchial aspirate	2018
**CM9174**	Bronchoalveolar lavage	2018
**CM9189**	Bronchial aspirate	2018
**CM9195**	Synovial Liquid	2018
**CM9228**	Sputum	2018
**CM9267**	Muscle Biopsy	2018
**CM9298**	Nasal Biopsy	2018
**CM9326**	Bronchoalveolar lavage	2018
**CM9329**	Cerebral Abscess	2018
**CM9331**	Bronchoalveolar lavage	2018
**CM9684**	Mastoid debridement	2019
**TP642**	Sputum (cystic fibrosis)	2017
**TP968**	Sputum (cystic fibrosis)	2018
**TP992**	Surgical wound	2018
**TP1004**	Sputum (cystic fibrosis)	2018
**TP1115**	Sputum (cystic fibrosis)	2018
**TP1179**	Sputum (cystic fibrosis)	2018

Supplementary Table 2. Primers used for *Aspergillus flavus* *cyp*51A, *cyp*51B and *cyp*51C DNA amplification and sequencing and RT-PCR from RNA.

Primer use	Primer name	Primer sequence (5'-3')	Reference
*cyp*51A gene and promoter	Afla_Cyp51A_F1	CAAGAACAGCCTGCACAGAG	[25]
	Afla_Cyp51A_R1	GGGTGGATCAGTCTTATTA	
	Afla_Cyp51A_F2	GCAATCATCGTCCTAAATC	
	Afla_Cyp51A_R2	CTGTCCATTCTTGTAGGTA	
	Afla_Cyp51A_F3	GCATGAGGGAGATCTATATG	
	Afla_Cyp51A_R3	CCTATAATTGCTGGTTTCG	
	Afla_Cyp51A_F4	TGAAGCTATTCAATGTAGAC	
	Afla_Cyp51A_R4	ACTGCTGATGGTGTGCTAAG	
RT_PCR	Cyp51A_RT_F	TAAATCTGTTGCGCCAGCTC	This work
	Cyp51A_RT_R	ATCTCCTCGGCATTGACATC	
**Primer use**	**Primer name**	**Primer sequence (5'-3')**	
*cyp*51B gene and promoter	Afla_Cyp51B_F1	ATGGGCATCCTAGCTGTCATTC	This work
	Afla_Cyp51B_R1	GGCGGTGTATATGGTAATCTC	
	Afla_Cyp51B_F2	CCCTTGGTATTTCATTGGTTCCC	
	Afla_Cyp51B_R2	TTTCATGTTACCATGGGCCC	
	Afla_Cyp51B_F3	TTTCATGTTACCATGGGCCC	
	Afla_Cyp51B_R3	TTCAGAGCTAACAGCGATGGC	
	Afla_Cyp51B_F4	CGGAAGAACATTTCCCTGATCC	
	Afla_Cyp51B_R4	CGTCTGGCAATATCATGCAC	
RT_PCR	Cyp51B_RT_F	CGGAAGTACCATCAGCTATG	This work
	Cyp51B_RT_R	ACACAGGAGTTGTAAGTGGG	
**Primer use**	**Primer name**	**Primer sequence (5'-3')**	
*cyp*51C gene and promoter	CypC_Af1_F	CAATGGTGCTGACAAACCTG	This work
	CypC_Af2_R	CAAAGGAGCGACACATAAG	
	AflaCYP51CF2	GGTAATGTCTGGTCATAGG	[25]
	AflaCYP51CR2	ATGAGCTTGGAATTGGG	
	AflaCYP51CF3	CGAATTCATCCTCAATGG	
	AflaCYP51CR3	GTCTCTCGGATCACATT	
	AflaCYP51CF4	GGAACTCTACCAAGAGCA	
	CypC_Af8_R	GCTCATCATAATGCATGAGG	
RT_PCR	Cyp51C_RT_F	CGCATGCTGAACAAGACTAG	This work
	Cyp51C_RT_R	TCCGCGTTCACGTCTTTCAG	

Supplementary Table 3. GenBank Accession Numbers of all *A. flavus cyp*51A, *cyp*51B and *cyp*51C.

Strain	*cyp*51s	GenBank Acc. Ns
CM7668	*cyp*51A	MT849811
CM8087	*cyp*51A	MT849810
CM8098	*cyp*51A	MT849809
CM9165	*cyp*51A	MT849808
CM9174	*cyp*51A	MT849807
CM9189	*cyp*51A	MT849806
CM9195	*cyp*51A	MT849805
CM9228	*cyp*51A	MT849804
CM9267	*cyp*51A	MT849803
CM9298	*cyp*51A	MT849802
CM9326	*cyp*51A	MT849801
CM9329	*cyp*51A	MT849800
CM9331	*cyp*51A	MT849799
CM9684	*cyp*51A	MT849798
TPH642	*cyp*51A	MT849797
TPH968	*cyp*51A	MT849796
TPH992	*cyp*51A	MT849795
TPH1004	*cyp*51A	MT849794
TPH1115	*cyp*51A	MT849793
TPH1179	*cyp*51A	MT849793
ATCC2004304	*cyp*51A	MT849812
CM7668	*cyp*51B	MT849771
CM8087	*cyp*51B	MT849772
CM8098	*cyp*51B	MT849773
CM9165	*cyp*51B	MT849774
CM9174	*cyp*51B	MT849775

CM9189	*cyp*51B	MT849776
CM9195	*cyp*51B	MT849777
CM9228	*cyp*51B	MT849778
CM9267	*cyp*51B	MT849779
CM9298	*cyp*51B	MT849780
CM9326	*cyp*51B	MT849781
CM9329	*cyp*51B	MT849782
CM9331	*cyp*51B	MT849783
CM9684	*cyp*51B	MT849784
TPH642	*cyp*51B	MT849785
TPH968	*cyp*51B	MT849786
TPH992	*cyp*51B	MT849787
TPH1004	*cyp*51B	MT849788
TPH1115	*cyp*51B	MT849789
TPH1179	*cyp*51B	MT849790
ATCC2004304	*cyp*51B	MT849791
CM7668	*cyp*51C	MT853225
CM8087	*cyp*51C	MT853226
CM8098	*cyp*51C	MT853227
CM9165	*cyp*51C	MT853228
CM9174	*cyp*51C	MT853229
CM9189	*cyp*51C	MT853230
CM9195	*cyp*51C	MT853231
CM9228	*cyp*51C	MT853232
CM9267	*cyp*51C	MT853233
CM9298	*cyp*51C	MT853234
CM9326	*cyp*51C	MT853235
CM9329	*cyp*51C	MT853236
CM9331	*cyp*51C	MT853237
CM9684	*cyp*51C	MT853238

TPH642	*cyp*51C	MT853239
TPH968	*cyp*51C	MT853240
TPH992	*cyp*51C	MT853241
TPH1004	*cyp*51C	MT853242
TPH1115	*cyp*51C	MT853243
TPH1179	*cyp*51C	MT853244
ATCC2004304	*cyp*51C	MT853245
